# The Potential Diagnostic Trap of Unexpected Intracranial Hyalohyplymycosis—A Case Report and Brief Literature Review

**DOI:** 10.3390/jcm14082594

**Published:** 2025-04-10

**Authors:** Ligia Gabriela Tataranu

**Affiliations:** 1 Department of Neurosurgery, Carol Davila University of Medicine and Pharmacy, 020021 Bucharest, Romania; ligia.tataranu@umfcd.ro; 2 Department of Neurosurgery, Bagdasar-Arseni Emergency Clinical Hospital, 041915 Bucharest, Romania

**Keywords:** intracranial aspergillosis, fungal infections, neurosurgery, immunocompetent

## Abstract

**Background:** Intracranial fungal infection represents a rare entity, especially in immunocompetent patients. The available neuroimaging methods may show nonspecific results; thus, a final diagnosis is based on histopathological examination. **Methods**: This report aims to describe a rare case of a young immunocompetent adult who developed a secondary skull base infection, probably due to primary sinonasal involvement. The patient was an 18-year-old immunocompetent male adult with intracranial hyalohyphomycosis, diagnosed and neurosurgically treated in the 3rd Neurosurgical Department of Clinical Emergency Hospital Bagdasar-Arseni. **Results**: The neurosurgical excision and intense antifungal therapy led to very good outcomes. The particularity of this case is highlighted by the local traumatic component due to boxing practice. The impact of presented cases of this kind is significant given the scarce information regarding this subject, and it can contribute to a better understanding of the underlying mechanisms of this pathology. Intracranial fungal infections are very rare and very often difficult to diagnose. Although it is known that their prognosis implies a mortality of up to 60% in immunocompromised patients, in immunocompetent cases, the exact characteristics of the disease, as well as the prognostic factors, are yet to be elucidated. **Conclusions**: The neuroimaging results are nonspecific in many cases, but a diagnosis is based on the histopathological results with specific features. Even though antifungal therapy is the main treatment for fungal infections, in cases of intracranial lesions, a diagnosis may be obtained after neurosurgical excision, and drug therapy will come second.

## 1. Introduction

Notwithstanding the fact that most fungi have a symbiotic relationship with humans, in certain favorable conditions, they can become harmful. In the central nervous system, besides meningitis and meningoencephalitis, fungi can determine a localized infection that can present as either a granuloma or an abscess [1]. The fungal granuloma results from an intense inflammatory process around the infected area, and some fungi can invade the vascular walls, leading to intracranial bleeding [2]. Although fungal infections are persistent in immunocompromised cases or patients with specific uncontrolled comorbidities, with a high mortality rate, they are extremely rare in immunocompetent individuals [3,4].

*Aspergillus* species are the most frequently incriminated pathogens in intracranial fungal granulomas, and risk factors are mainly represented by malnutrition; treatment with antitubercular drugs, antibiotics, and steroids; and parasitic infections [5]. Other causative reported fungi are represented by *Cryptococcus neoformans*, *Candida* species, *Zygomycetes*, melanized fungi, and other fungal pathogens such as *Histoplasma capulatum*, *Peudoallesheria boydii*, or *Maduralla mycetomatis* [6]. Although the majority of these fungi can cause infections in patients with certain predisposing conditions, pathogenic agents like *Cryptococcus*, *Coccidioides*, or *Histoplasma* can affect immunocompetent patients as well [7].

Regarding the routes of contamination, it has been stated that this infection can occur as a dissemination from a distant infection, as well as through the blood circulation or as an extension from a neighboring area, although the latter is very rare [8] and constitutes the subject of the current article. A direct infection has also been described in the medical literature [8].

A diagnosis is based on a histopathological assessment given that the clinical findings and neuroimaging examinations are not specific, and prompt treatment with antifungal medicines is the main recommendation [9]. Some authors suggest that the best treatment option is represented by radical surgery and intensive antifungal administration [10,11].

## 2. Case Presentation

### 2.1. The Diagnosis Timeline (Figure 1)

An 18-year-old male suffered a minor head injury and was examined in the emergency department of another hospital, where a native brain CT scan was immediately performed. Although the results showed no post-traumatic brain injuries, a sellar and suprasellar mass was incidentally diagnosed, and a contrast-enhanced brain MRI was then suggested.

**Figure 1:**
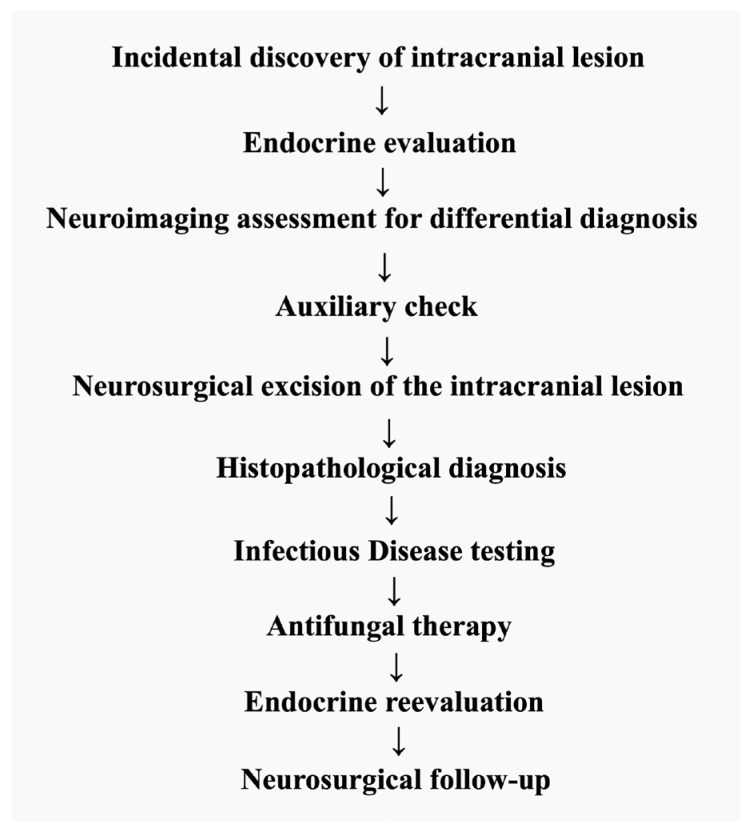
Timeline of events in the present case report.

The patient underwent the recommended neuroimaging examination, which showed a suprasellar mass lesion extending into the superior part of the sella and anteriorly over the planum sphenoidale, with a polylobulated aspect, a mixed appearance, and solid, cystic, and hematic components, measuring 25/32/23 mm (transversal/craniocaudal/anteroposterior).

**Figure 2:**
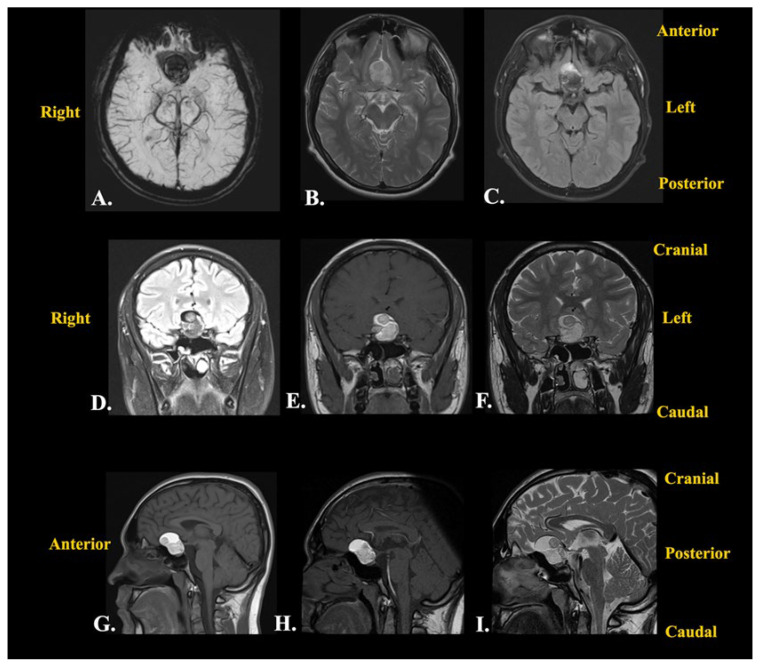
Preoperative contrast-enhanced brain MRI performed before the patient’s admission into our department: (**A**) axial susceptibility-weighted imaging sequence; (**B**) axial T2-weighted imaging Fast or Turbo Spin Echo (FSE/TSE) sequence; (**C**) axial Transverse Relaxation Time (T2)-weighted imaging TSE sequence using dark fluid; (**D**) coronal T2-weighted dark fluid sequence; (**E**) coronal T1-weighted dynamic contrast-enhanced sequence; (**F**) coronal T2-weighted TSE sequence; (**G**) sagittal T1-weighted TSE sequence; (**H**) sagittal T1-weighted contrast-enhanced sequence; (**I**) sagittal T2-weighted TSE sequence.

The solid (tisular) component of the mass lesion showed intense gadolinium enhancement without restricted diffusion. Its neighboring structures were represented by the sphenoid sinus (anterior and inferior), the carotid arteries and cavernous sinuses, without invading them (lateral), and a displaced optic chiasm and anterior cerebral arteries (posterior), as well as the optic nerves (lateral). Furthermore, the sella turcica was enlarged, and the hypophyseal gland measured 18/7/12 mm (transversal/craniocaudal/anteroposterior), with a flattened superior contour due to the mass lesion. The paranasal sinus mucosa was thickened, and a right lateral deviation of the nasal septum was observed (Figure 2).

Given the rising suspicion of a pituitary neuroendocrine tumor (PitNET), the patient was referred to the endocrinological department, where he was diagnosed with a corticotroph deficiency, and cortisol replacement treatment was initiated, with the administration of hydrocortisone 10 mg daily, divided into two doses.

Finally, after all the aforementioned assessments, the patient came for a neurosurgical examination and was admitted to the 3rd Neurosurgical Department from Clinical Emergency Hospital Bagdasar-Arseni 3 months after the initial diagnosis, with no symptoms. Regarding his medical history, the patient underwent strabismus surgery in childhood and had a COVID-19 infection 3 years earlier. Moreover, he started practicing boxing in childhood and had experienced multiple nasofacial traumas.

### 2.2. Neuroimaging Assessment

**Figure 3:**
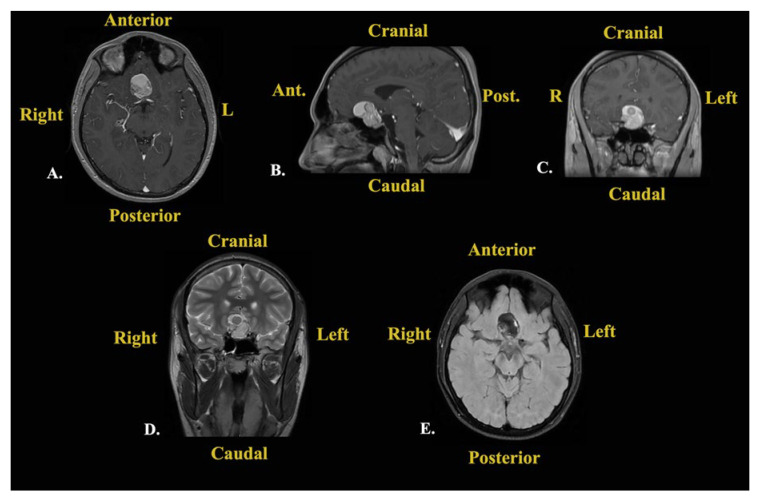
Preoperative contrast-enhanced brain MRI: (**A**) axial T1 contrast-enhanced Fast Spoiled Gradient-Echo (FSPGR) sequence; (**B**) sagittal contrast-enhanced T1-weighted multiplanar reformation/reconstruction (MPR) sequence; (**C**) coronal contrast-enhanced T1-weighted MPR sequence; (**D**) coronal T2-weighted FSE sequence; (**E**) axial T2-weighted FLAIR FS sequence.

Given the amount of time that had passed since the initial discovery of the intracranial lesion (3 months), contrast-enhanced MRI was repeated (Figure 3). The results showed a small lesional growth, and the new dimensions described were 30.7/34/24 mm (transversal/craniocaudal/anteroposterior). The mass lesion had an inhomogeneous structure due to the presence of a solid component with moderate enhancement, a hemorrhagic component, and a liquid component with high levels of protein. The hypophyseal gland was declivitous and flattened from the superior view, while the optic chiasm was slightly pushed from the posterior and superior views.

**Figure 4:**
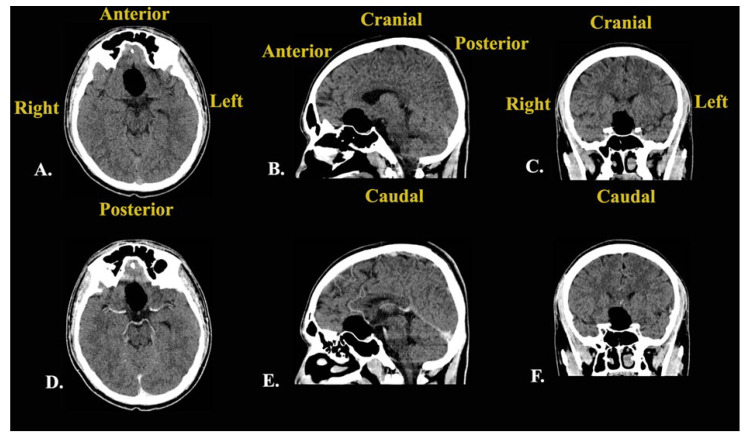
Preoperative brain CT scan: (**A**) native axial sequence; (**B**) native sagittal sequence; (**C**) native coronal sequence; (**D**) contrast-enhanced axial sequence; (**E**) contrast-enhanced sagittal sequence; (**F**) contrast-enhanced coronal sequence.

Following this investigation, a brain CT scan was performed to obtain more imagistic details (Figure 4).

The results depicted a tumoral mass, well delimited and localized at the median line in the suprasellar cistern and without a surrounding edema, without contrast enhancement, and with intrasellar prolabation. The lesion had a slightly inhomogeneous structure given the presence of areas with high densities (−27 UH, compared to −116 UH), corresponding to microcalcifications/hemorrhages/saponification of fats.

Subsequent cerebral angiography excluded any intracranial vascular malformations and highlighted the relationships with the neighboring blood vessels. An intracranial non-enhancing compressive mass lesion was determined, with a bilateral A1 segment of the anterior cerebral artery’s ascension (a positive “vault sign”).

After these investigations, the differential diagnoses comprised first and foremost an intracranial dermoid cyst given the hyperintense aspect in the T1-weighted sequence and the variable aspect in the T2-weighted sequence on MRI, as well as the well-defined low-attenuation and non-contrast-enhanced aspect in the CT scan [12]. The second option was craniopharyngioma given the hyperintense aspect in the T1-weighted sequence, with vivid enhancement, and the variable aspect in the T2-weighted sequence on MRI [13]. However, the CT scan aspect was not necessarily compatible with this type of lesion [13]. The third option was represented by a PitNET with intratumoral apoplexy given the moderate enhancement in the T1-weighted sequence and the variable aspect in the T2-weighted sequence on MRI [14]. However, the CT scan images were incompatible with this suspected lesion. The fourth option was a teratoma due to the hyperintense aspect and protein-rich fluid’s moderate enhancement in the T1-weighted sequence and the variable aspect in the T2-weighted sequence [15]. Nevertheless, the CT scan images did not suggest this type of lesion.

### 2.3. The Auxiliary Check

The routine complete blood count at admission revealed normal values. The results showed no abnormality in terms of biochemistry, coagulation, C-reactive protein, or urine. His ophthalmic results were normal, and the ENT examination determined the thickening of the paranasal sinus mucosa and the right lateral deviation of the nasal septum. The chest radiograph was normal, while a plain radiograph of the sella turcica revealed a slight enlargement in this structure.

### 2.4. The Neurosurgical Treatment

**Figure 5:**
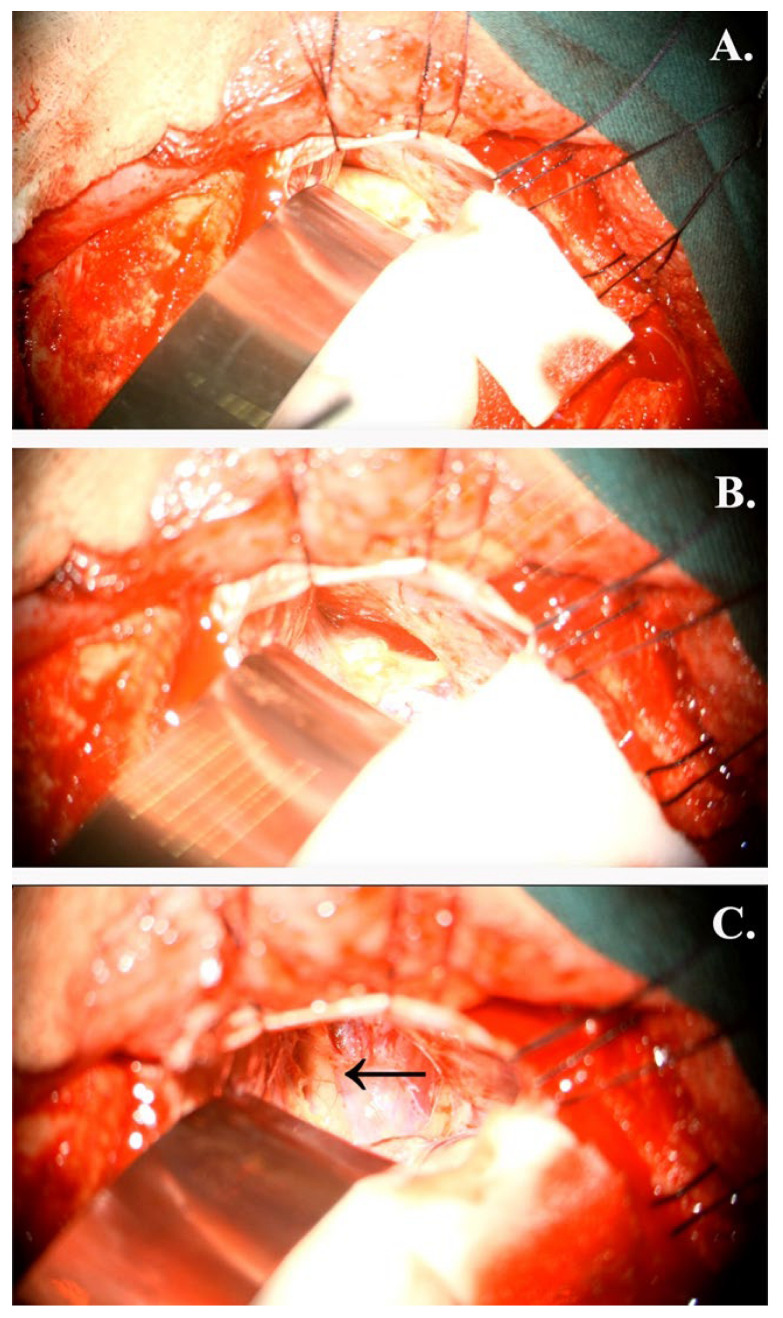
Intraoperative microscopic aspect of the intracranial fungal mass: (**A**) an image after the dural opening depicting a heterogeneous yellowish-white mass; (**B**) an image after partially draining out the cystic fluid and before complete excision; (**C**) multiple adherences, specifically to the optic nerve (black arrow), even after complete resection.

After the preoperative evaluation, a neurosurgical intervention was decided upon, and the patient gave his written consent. Thus, a gross total resection was performed through a transcranial right frontobasal transdural approach under general anesthesia. During the operation, various membranes were discovered in the subarachnoid space, adherent to the neighboring structures, specifically to the optic nerves. Therefore, they were dissected as much as possible. Following the dissection, a yellowish-white lesion was found, with a soft consistency and a cystic component (Figure 5). The tumoral mass had clear boundaries and a capsule.

**Figure 6:**
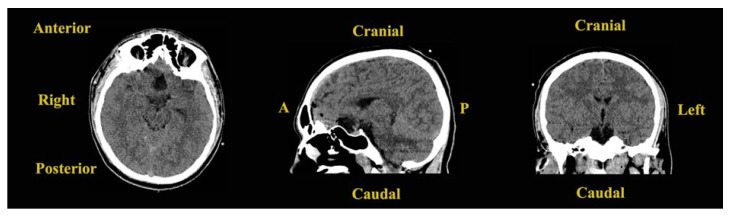
Postoperative native brain CT scan shows a normal aspect (A-anterior; P-posterior).

Following the neurosurgical intervention, the patient underwent the usual postoperative treatments. In addition, a postoperative cerebral CT scan confirmed the gross total resection (Figure 6).

In the short term, the local and neurological evolution of the patient was favorable, and he was discharged without any symptoms or clinical manifestations. He was advised to continue his substitutive corticosteroid therapy until the endocrinological check-up and to come back for a reexamination after 3 months or if ever a new local or neurological symptom appeared.

**Figure 7:**
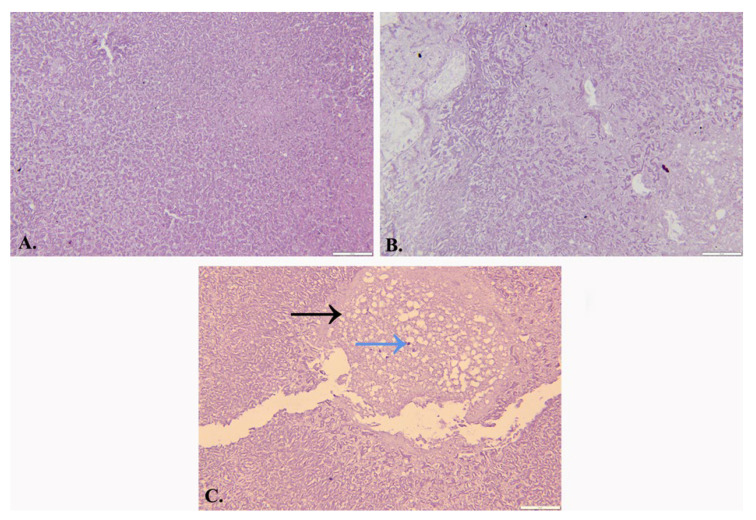
Photomicrographs depicting histopathologic features specific to intracranial aspergillosis: (**A**) Hematoxylin–Eosin staining under 40× magnification showing abundant fungal hyphal branching, suggesting *Aspergillus* species; (**B**) Hematoxylin–Eosin staining under 40× magnification showing granuloma and giant cells with fungal hyphae; (**C**) periodic acid–Schiff stain under 40× magnification showing necrotizing granulomatous inflammation (black arrow) and multinucleated giant cells with narrow fungal hyphae inside (blue arrow), consistent with *Aspergillus* species.

After one week, the histopathological examination determined the diagnosis of intracranial aspergillosis (Figure 7).

**Figure 8:**
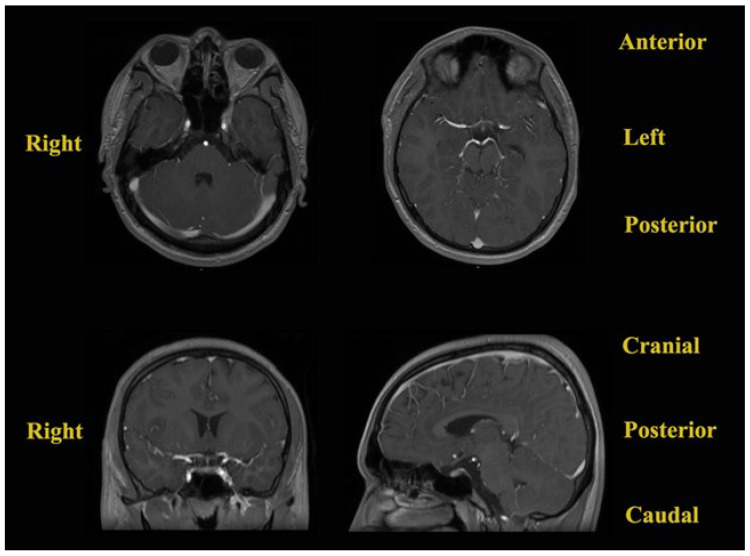
Contrast-enhanced brain MRI (T1-weighted sequences) performed at the 3-month follow-up.

The patient was reevaluated by the endocrinological department at 3 months. Still, his corticotroph deficiency had persisted, and the patient was advised to continue his substitutive corticosteroid treatment at the same doses and in the same manner. At the 3-month follow-up, contrast-enhanced brain MRI was performed, which showed no recurrences or abnormalities (Figure 8).

## 3. Discussion

Intracranial fungal infections have become more frequent in recent decades, especially due to the administration of long-term immunosuppressive treatments [16]. *Aspergillus* species represent the most commonly responsible pathogens, entering the central nervous system either through the bloodstream or through a contiguous spread from neighboring structures (e.g., the paranasal sinuses, the ears). This ubiquitous fungus is frequently found in soils and plants, it produces spores that are inhaled and contaminate the lungs or the paranasal sinuses, and it usually has septate hyphae [17].

The symptoms are unspecific, without fever, while the neuroimaging results are similar to those for mass lesions [16,18]. In this case report, the primary site of infection involved was considered to be represented by the sinonasal region, with secondary intracranial involvement. Multiple nasofacial traumas due to boxing, the thickening of the sinus mucosa, and deviation of the nasal septum were predisposing factors supporting this theory.

The described infection can have an acute, subacute, or chronic course, with a longest recorded course of 20 years [19].

In the scarce medical literature regarding intracranial aspergillosis, the mortality rate is very high, between 60 and 100% [20]. Differently from other pathogens, *Aspergillus* can invade the vascular walls and lead to hemorrhages or infarcts [21]. A hemorrhagic component is present in up to 25% of cases, and the contrast enhancement is minimal in immunocompromised cases, while it is stronger in immunocompetent ones [22]. The presented case also had a hemorrhagic component, as well as a moderate enhancement in MRI.

The risk factors for intracranial aspergillosis are represented by HIV infection, hematologic diseases and their treatment [23], solid organ transplants [24], and corticosteroid use [25]. It has been stated that the latter factor can impede the macrophages and monocytes from killing *Aspergillus*, even after short-term corticosteroid administration [25]. Moreover, the intranasal use of corticosteroids has also been described as a risk factor [26].

In the presented case, the patient performed boxing starting in childhood, which resulted in repeated nasofacial traumas. Given that the nasal mucosa is a major immunity component, any trauma may disrupt immune homeostasis and the immune barrier while supporting the invasion of pathogens [27]. Thus, I consider this personal context to be a predisposing factor for intracranial aspergillosis. Cases of central nervous system aspergillosis secondary to traumatic events have previously been described in the literature, although they are extremely rare [28,29,30].

A recent study by Bhuskute et al. aimed to assess the etiological and anatomical factors involved in fungal infections that affect the anterior skull base and concluded that COVID-19 infection and diabetes mellitus were the main incriminated factors [31]. The presented patient had experienced a COVID-19 infection 3 years earlier. In 2022, Bhandari et al. reported COVID-19-associated aspergillosis in 92 Indian patients over 6 months [32]. All of the included patients were therapeutically managed using surgical excision and antifungal therapy, and no deaths were recorded.

Although an early diagnosis is important in this disease, it can be very challenging in many cases [33] and can be mistaken for other intracranial infections or space-occupying lesions [34,35,36]. The neuroimaging features are very often nonspecific [37], even in immunocompetent patients [38]; thus, histopathological confirmation is needed.

A differential diagnosis was made of an intracranial dermoid cyst considering the hyperintense aspect in the T1-weighted sequence and the variable aspect in the T2-weighted sequence on MRI. Furthermore, the well-defined, low-attenuated, and non-contrast-enhanced aspect in a CT scan of a dermoid cyst [12] was quite similar to the presented patient’s lesion. I even considered this option after seeing the intraoperative aspects. The second differential diagnosis made was craniopharyngioma considering the hyperintense aspect in the T1-weighted sequence and the variable aspect in the T2-weighted sequence on MRI [12]. However, craniopharyngiomas are vividly enhanced, while the presented lesion was moderately enhanced in the MRI. Moreover, the CT scan aspect was not necessarily compatible with this type of lesion [13]. Another option for the differential diagnosis was represented by a pituitary macroadenoma with intratumoral apoplexy. This diagnosis was supported by the moderate enhancement in the T1-weighted sequence and the variable aspect in the T2-weighted sequence on MRI [14], although the CT scan aspect of this type of lesion was not compatible with the patient’s lesion. Another differential diagnosis was represented by a teratoma given the hyperintense aspect and protein-rich fluid with moderate enhancement in the T1-weighted sequence and the variable aspect in the T2-weighted sequence [15]. Although they were less likely, I considered differential diagnoses of a Rathke’s cleft cyst, an optic pathway glioma, a meningioma, metastasis, an ectopic germinoma, or a giant aneurysm.

The detection of galactomannan antigens in the cerebrospinal fluid (CSF), which are specific to aspergillosis, can assist in a diagnosis [39]. In this case report, the patient was tested further by an Infectious Disease doctor after surgery, but his serum galactomannan antigen results were negative. HIV and hepatitis were excluded, and antifungal therapy was administered.

Although Kattner et al. discussed in their article the option of next-generation sequencing, which can identify whether the pathogenic agent is in the CSF or plasma and can significantly increase the chances of a prompt diagnosis [40], in the current case, this method was not available. Although the histopathological examination of the present patient concluded with a diagnosis of intracranial aspergillosis, no mycologic studies confirmed this specific diagnosis. Galactomannan determination was performed but with negative results, and there was no culture or antibody testing to support the histopathological diagnosis. Thus, the final diagnosis considered was hyalohyplymycosis. In histological preparations, most hyalohyphomycetes are seen in the same way (*Aspergillus*, *Fusarium*, *Scedosporium*, et cetera), and some phaeohyphomycetes are even difficult to detect without special stains for melanin, such as Masson–Fontana staining [41].

The therapeutic options for this disease are represented by antifungal drugs and neurosurgical treatment, as well as symptomatic treatment [42]. Furthermore, intracavitary amphotericin B administration was reported by Kural et al., with debatable results [43].

Notwithstanding the fact that the immunopathogenesis of intracranial fungal infections is yet to be fully understood, in 2007, Dotis and Roilides stated that interaction between activated brain-resident cells and the expression of immunoenhancing and immunosuppressing cytokines and chemokines may partially explain the process [44].

Haran and Chandy reported 13 cases of immunocompetent histopathologically confirmed intracranial aspergillosis over 12 years [45]. The mean reported age was 35.4 years, and the clinical presentations were typical for an intracranial mass. After being treated with partial excision/biopsy and amphotericin B, Rifampicin, and 5-flurocytosine (5FC), three patients died, while one was lost to follow-up.

To date, no immunodeficiencies have been diagnosed regarding the immunocompetence status of the presented patient. Pianetti et al. discuss the matter of immunodeficiency [46], stating that such a condition can be diagnosed throughout one year of follow-up if at least one of the following clinical alterations are found: (1) more than two systemic bacterial infections (septicemia, meningitis, osteomyelitis); (2) three severe respiratory infections (pneumonia, sinusitis) or three bacterial infections (cellulitis, otitis media with effusion, lymphadenitis); (3) infection in an unusual location (a hepatic or cerebral abscess); (4) infections with less frequent pathogens (pulmonary aspergillosis, disseminated candidosis or *Serratia marcescens* infection, *Nocardia* species, and *Berkoldaria cepacian*); and (5) very serious infections [46].

Sundaram et al. reported a series of 73 cases of central nervous system aspergillosis over 17 years, with a mean age of 38 years [47]. Regarding the route of contamination, the authors reported the sinocranial route in 38 cases, the sino-orbitocranial route in 8 cases, the sino-orbital route in 8 cases, the orocranial route in 4 cases, the hematogenous route in 7 cases, and an uncertain route in 8 cases. None of these patients were positive for HIV, and risk factors were identified in 12% of cases. The authors stated that the most frequent geographical areas reporting intracranial aspergillosis were India, Sudan, Saudi Arabia, and Pakistan. The majority of reported cases of histoplasmosis, coccidioidomycosis, and blastomycosis have been from the United States given the preponderance of these pathogens in this geographical area [48]. Regarding the geographical question, it is worth discussing a few pieces of information concerning *Aspergillus* and the main fungi in Romania. It has been stated that in immunocompromised patients, the annual incidence of invasive aspergillosis is 458 cases and the prevalence is 7.7/100,000, while the prevalence of pulmonary aspergillosis after pulmonary tuberculosis is 8.98 per 100,000 cases. Furthermore, the prevalence of allergic bronchopulmonary aspergillosis comprises 29,397 cases yearly, with more than 2% of Romanians suffering from a severe fungal infection [49]. Fungal strains from the air in different areas adjacent to bakeries have been reported, with *Aspergillus*, *Penicillium*, *Alternaria*, *Fusarium*, *Ulocladium*, *Neurospora*, and *Trichoderma* identified [50]. Moreover, Aspergillus strains have also been detected in cereals in south-western Romania [51]. Cases of resistant fungal strains isolated from Romanian vineyard soil samples were also reported in a recent article from 2023, identifying *Aspergillus* section *Nigri*, *Aspergillus* section *Usti*, *Penicillium* species, and *Mucorales* [52].

Siddiqui et al. reported a series of 25 cases of craniocerebral aspergillosis of sinonasal origin in immunocompetent patients, with a mean age of 36.5 years [10]. All patients were treated with a subtotal excision and oral antifungal drugs (itraconazole) +/− intravenous amphotericin B, and the results confirmed a mortality rate of 28%.

Denning et al. reported a total of 1223 cases of invasive aspergillosis, and cerebral aspergillosis had a reported mortality rate of 99% [53].

Alrajhi et al. reported 23 cases of intracranial aspergillosis in immunocompetent hosts over 7 years, with a mean age of 25 years [54]. All patients, except for five of them, received antifungal therapy after surgical excision, and those who did not receive this relapsed. Only one patient died, while one was lost to follow-up.

Murthy et al. presented a retrospective study including 21 cases of central nervous system aspergillosis, of whom 16 had sino-cranial involvement, with a mean age of 41.8 years [55]. Many authors have considered diabetes mellitus to be a predisposing factor, and this study supports this aspect. The therapeutic management comprised antifungal treatment (amphotericin B with or without 5FC) and surgical intervention in all patients. The mortality rate was 37.5% following postoperative dissemination and recurrence.

Dubey et al. reported 40 cases of intracranial aspergillosis over 22 years, with a mean age of 40.2 years [5]. The therapeutic management included surgical excision and antifungal drugs. However, the mortality rate was 63%.

Considering the chronological aspect as the main feature, between 1976 and 2018, 182 immunocompetent patients with intracranial aspergillosis were reported by Sung et al., with a median age of 40 years and vascular complications in 26.3% [56]. Although the mortality rates significantly improved over time, this study determined a rate of 31.9% deaths, while 44.5% reported good outcomes.

In 2019, Bora et al. reported 18 immunocompetent patients diagnosed with intracranial aspergillosis and treated using surgical excision and antifungal treatment [57]. This study determined a mortality rate of 44%, with six patients becoming disease-free.

As in the present case report, intracranial aspergillosis affecting the sellar and parasellar region lesions was previously described by Zhang et al. in four cases treated using surgical excision and antifungal medication, with very good outcomes in three patients, as one patient was lost to follow-up [58].

Intracranial complications of aspergillosis, such as mycotic aneurysms, have been described in the literature; however, their outcomes have not been favorable, mostly due to fatal subarachnoid hemorrhages [59,60,61,62,63], with very few exceptions [64,65]. Furthermore, a rare case of the co-existence of *Nocardia farcinica*, *Pneumocystis jirovecii*, and *Aspergillus fumigatus* was recently reported by Jinlin et al. in a 62-year-old mildly immunosuppressed male. Nevertheless, the patient improved after receiving meropenem (later replaced with imipenem), compound sulfamethoxazole, linezolid, ganciclovir, omeprazole, and intravenous voriconazole, with full recovery. The authors concluded that if during treatment with the antifungal voriconazole, the trough concentration does not reach the target, the administration of omeprazole will help to increase its concentration [66].

Cerebral infarction due to *Aspergillus thrombus* was reported by Katano et al. in 2022 in an 88-year-old man, with re-occlusion after mechanical thrombectomy [67].

Cases of intracranial aspergillosis in pregnant women have also been described, with both unfavorable [68] and favorable results [69].

Despite significant advances in recent decades related to central nervous system aspergillosis, the registered mortality rates are still high. Notwithstanding, a prompt diagnosis is an essential factor for favorable outcomes. This can be very difficult given the versatility of this disease and the nonspecificity of its symptoms and neuroimaging aspects. New therapeutical agents and combination drug therapies are currently being developed, providing promising results in the fight against central nervous system aspergillosis [70], and further studies are encouraged in order to fully uncover the mechanisms of its appearance and development.

## 4. Conclusions

Intracranial fungal infection by *Aspergillus* species should be considered in patients with sellar and parasellar lesions as a differential diagnosis when the neuroimaging results are nonspecific. The best therapeutic management comprises a thorough examination of the clinical, anamnestic, neuroimaging, and histopathological results. Although the main therapeutic option is represented by intensive antifungal therapy, neurosurgical excision of the intracranial mass should be considered in selected cases and sometimes is the only viable option, as it was in the current case when a different initial diagnosis was reached. Nevertheless, intracranial fungal infections may always be a surprise to doctors, and further studies are needed to understand its complete underlying processes better.

## Data Availability

The data presented in this study are available on request from the corresponding author for legal reasons.
